# Network Toxicology and Molecular Docking Analysis of Tetracycline-Induced Acute Pancreatitis: Unveiling Core Mechanisms and Targets

**DOI:** 10.3390/toxics12120929

**Published:** 2024-12-21

**Authors:** Hang Lei, Yimao Wu, Wenjun Ma, Jiaqi Yao, Pengcheng Zhang, Yong Tian, Yuhong Jiang, Zhijun Xie, Lv Zhu, Wenfu Tang

**Affiliations:** 1 West China Center of Excellence for Pancreatitis, Institute of Integrated Traditional Chinese and Western Medicine, West China Hospital, Sichuan University, Chengdu 610041, China; leihang@stu.scu.edu.cn (H.L.); yaojq@stu.scu.edu.cn (J.Y.); zpc1113@stu.scu.edu.cn (P.Z.); tianyscuhx@163.com (Y.T.); jocelynjiang@stu.scu.edu.cn (Y.J.);; 2 Second Clinical Medical College, Guangdong Medical University, Dongguan 523808, China; yimaowu_doctor2024@163.com; 3 Stomatological Hospital of Chongqing Medical University, Chongqing Medical University, Chongqing 401147, China; ma-wenjun@foxmail.com

**Keywords:** tetracycline, acute pancreatitis, network toxicology, molecular docking, toxicant metabolism

## Abstract

Acute pancreatitis (AP), induced by tetracycline, a widely used antibiotic, poses significant clinical and toxicological challenges, yet its molecular mechanisms remain unclear. This study aims to promote drug toxicology strategies for the effective investigation of the putative toxicity and potential molecular mechanisms of antibiotic drugs through the study of tetracycline in AP. Using the SwissTargetPrediction, SEA Search, Super-PRED, GeneCards, Drugbank, Online Mendelian Inheritance in Man (OMIM), and Therapeutic Target Database (TTD), we identified 259 potential targets associated with tetracycline exposure and AP. Further refinement via the STRING database and Cytoscape (version 3.10.1) software highlighted 22 core targets, including TP53, TNF, and AKT1. Functional enrichment via the Database for Annotation, Visualization, and Integrated Discovery (DAVID) identified pathways through Gene Ontology (GO) terms and the Kyoto Encyclopedia of Genes and Genomes (KEGG) database, highlighting PI3K-Akt, MAPK, HIF-1, and AGE-RAGE as critical mediators in tetracycline-induced AP. Molecular docking confirmed the strong binding between tetracycline and the core targets. Overall, these findings suggest that tetracycline may affect the occurrence and progression of pancreas-related inflammation by regulating pancreatic cell apoptosis and proliferation, activating inflammatory signaling pathways, and regulating lipid metabolic pathways. This study provides a theoretical basis for understanding the molecular mechanism of tetracycline-induced AP and lays the foundation for the prevention and treatment of digestive system diseases associated with excessive exposure to tetracycline antibiotics and certain tetracyclines. In addition, our network toxicology approach has accelerated the elucidation of toxic pathways in antibiotic drugs that lack specific characteristics.

## 1. Introduction

The antibiotic tetracycline, which has attracted much attention in medical use, is an important factor in inducing pharmacogenetic acute pancreatitis; however, its induction mechanism has not been fully clarified [1]. Traditional research methods are difficult to analyze the complex pathological process because they focus on a single index or local pathology, ignoring the organism’s overall internal environment and complex physiological pathways [2]. Tetracycline-induced acute pancreatitis is realized by affecting multiple physiological systems and complex molecular networks; therefore, new methods are needed to assess its potential health threats, so as to guide the rational use of clinical medication and the prevention and treatment of related diseases.

Acute pancreatitis is a common digestive disease with a variety of clinical manifestations, ranging from mild to severe, and if early treatment is not timely, it can develop into a severe and life-threatening condition [3]. Its etiology is diverse, and includes drug pancreatitis. Although the incidence of drug acute pancreatitis is low, it is on the rise with the increase in specific populations, it lacks specific manifestations, and its pathogenesis is still unclear, which may be related to allergies, drug cytotoxicity, sphincter contraction, or toxic metabolite accumulation [4,5,6].

Tetracycline-induced pancreatitis may be triggered by hypertriglyceridemia through the inhibition of protein synthesis, which results in the accumulation of defective proteins clogging up the pancreas [7,8]. Studies have shown that tetracycline-induced acute pancreatitis is strongly associated with underlying liver disease, with a longer time to acute pancreatitis with tetracycline in those without a history of liver disease and a shorter time required in those with severe liver dysfunction [9,10,11]. In addition, tetracycline has a direct effect on pancreatic cells, interfering with trypsinogen synthesis and affecting trypsin activity, which in turn increases the risk of morbidity [12].

The combination of network toxicology and molecular docking is a highly promising research strategy [13]. Network toxicology allows for the construction of relational networks and the translation of complex mechanisms into graphical models for easy analysis and prediction [14]. In this paper, we adopt this method to study the potential toxicity and mechanism of tetracycline in depth, and explore the toxicity pathway of tetracycline in acute pancreatitis through network toxicology, which is in line with the modern toxicity testing paradigm, and helps to elucidate the toxicological characteristics of tetracycline, predict the potential toxicity and molecular mechanism, provide ideas for the assessment of drug toxicity strategies, lay the foundation for the diagnosis of related diseases, and assist in the development of targeted interventions to minimize the toxic side effects.

## 2. Materials and Methods

**Figure 1:**
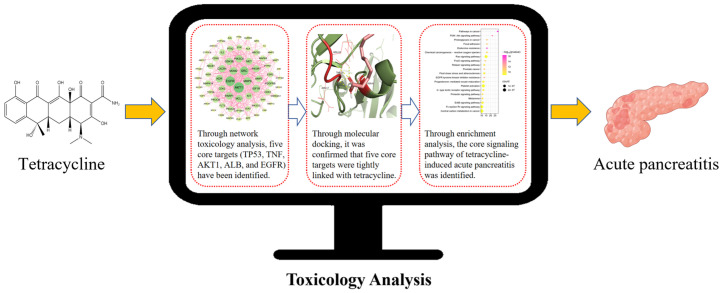
The flowchart of this study. TP53, tumor protein p53; TNF, tumor necrosis factor; AKT1, AKT serine/threonine kinase 1; ALB, albumin; EGFR, epidermal growth factor receptor.

We retrieved tetracycline action targets and acute pancreatitis disease targets from publicly available databases and identified common potential targets using Venn diagram analysis. These shared targets were input into the STRING database to construct a protein–protein interaction (PPI) network, which was then parametrically analyzed and visualized using Cytoscape (version 3.10.1) software to generate a PPI network diagram and identify key targets. Subsequently, Gene Ontology (GO) functional analysis and Kyoto Encyclopedia of Genes and Genomes (KEGG) pathway enrichment analysis of the potential and core targets were conducted using the DAVID database. The results identified tumor protein p53 (TP53), tumor necrosis factor (TNF), AKT serine/threonine kinase 1 (AKT1), albumin (ALB), and epidermal growth factor receptor (EGFR) as key targets, with lipid metabolism, advanced glycation end-products–receptor for advanced glycation end-products (AGE-RAGE), phosphatidylinositol-3-kinase/protein kinase B (PI3K-Akt), mitogen-activated protein kinase (MAPK), and hypoxia-inducible factor 1 (HIF-1) among the critical signaling pathways. Molecular docking further confirmed a strong association between tetracycline and these key targets. The workflow for this study is outlined in Figure 1.

### 2.1. Preliminary Network Analysis of Tetracycline Toxicity

The integration of network-based search algorithms and biotoxicity prediction methods into a relevant software tool allowed us to use structural modeling to predict toxicity associated with tetracycline compounds. Using ProTox-3.0 as a preliminary screening tool [15], we attempted to obtain basic but accurate information about tetracycline-induced toxicity.

### 2.2. Collection of Tetracycline Targets

The standard structure and SMILE node of tetracycline was determined by searching for “tetracycline” in the PubChem database [16]. Based on the search results using the keyword “tetracycline”, potential tetracycline targets were obtained from databases such as SwissTargetPrediction, SEA Search, and Super-PRED, and the species range was narrowed down to the “Homo sapiens predicted probability value” [17]. All targets were greater than 0.1. Finally, we integrated and de-emphasized the targets obtained above to generate a tetracycline target library.

### 2.3. Selection of Acute Pancreatitis-Related Target Network

With “acute pancreatitis” as the keyword, we comprehensively searched the domestic and international literature as well as the GeneCards, Drugbank, and OMIM databases to find related targets. In order to ensure the high correlation between the obtained genes and acute pancreatitis, we further processed the data obtained from the GeneCards database, set the “score” threshold as the median, and selected the genes with “score” values higher than the median to establish the acute pancreatitis target network (the Drugbank and OMIM databases do not give “scores” and have a small number of targets, so they were not processed). In addition, we used Venn diagrams to screen for common potential targets between tetracycline targets and acute pancreatitis targets, and identified intersections as potential targets for tetracycline-induced acute pancreatitis.

### 2.4. Construction of Protein Interaction Network and Screening of Major Targets

Scattered genes of potential targets of tetracycline-induced acute pancreatitis were entered into the STRING database. By restricting the species to “Homo sapiens”, setting the “Minimum Required Interaction Score” to “High Confidence > 0.7”, and selecting “FDR Strictness” to “0.7”, the STRING database was used to identify the potential targets of tetracycline-induced acute pancreatitis. These parameters ensured that we analyzed the active target protein corresponding to the target gene.

The results generated by STRING were then imported into the visual network biology analysis application Cytoscape (version 3.10.1) software, which calculates the parameters of each node in the network graph and displays the molecular connections [18]. This makes it possible to calculate the topological properties of network nodes and edges to generate PPI network graphs. The screening criteria for the primary target were as follows: select the node corresponding to the target that also satisfies the following conditions as the primary target for tetracycline-induced acute pancreatitis: (i) median centrality index > median, (ii) proximity centrality > median, and (iii) mean shortest path length > median.

### 2.5. Gene Function Analysis and Target Protein Pathway Enrichment

In order to investigate the biological functions of potential targets of tetracycline-induced acute pancreatitis, data were collected for GO analysis and KEGG pathway enrichment analysis using the DAVID database. We performed GO analyses including assessments of biological processes (BPs), cellular components (CCs), and molecular functions (MFs) to elucidate their main biological functions [19]. In addition, KEGG enrichment analysis was performed to identify important pathways associated with potential targets of tetracycline acute pancreatitis by setting the FDR threshold at <0.05 and identifying the main toxicity pathways that gained access to the targets [20].

In addition, we performed KEGG enrichment analysis of the core targets of tetracycline-induced acute pancreatitis using the DAVID database. The aim of this study was to further explore the pathways associated with acute pancreatitis through the main targets in order to elucidate and highlight the important signaling pathways involved in the biological process. Finally, we used the Microbiotics platform for visualization and analysis to effectively interpret and present the results of GO and KEGG analyses.

### 2.6. Molecular Docking of Tetracycline with Major Targets

This study employed molecular docking to analyze the interactions between tetracycline and the identified core target proteins in detail. The molecular structure of tetracycline was obtained from the PubChem database, and the crystal structures of 22 core proteins were retrieved from the RCSB Protein Data Bank (PDB). Protein structures in SDF format were imported into PyMOL (version 3.1.0) for preprocessing, including dehydration, and exported in mol2 format. These were subsequently imported into AutoDock Tools (version 1.5.7), hydrogenated, and saved in pdbqt format.

The small molecule ligand, converted to mol2 format via PyMOL, underwent hydrogenation and charge assignment, then was exported in pdbqt format. The docking area was defined as a blind docking grid using AutoGrid and AutoDock Tools, which generated PDB- and DLG-format files. The docking results were visualized and analyzed using PyMOL, with adjustments to the color scheme for clarity.

The interaction types analyzed included hydrogen bonding, van der Waals forces, hydrophobic interactions, electrostatic interactions, and π–π stacking interactions. The parameters used for these interactions were as follows: hydrogen bond lengths ranged from 0.15 to 0.30 nm, van der Waals radii summed to 0.05–0.10 nm, hydrophobic interaction distances ranged from 0.3 to 0.6 nm, electrostatic interaction distances ranged from 0.2 to 0.5 nm, and π–π stacking interaction distances ranged from 0.3 to 0.6 nm.

## 3. Results

### 3.1. Preliminary Network Assessment of Tetracycline Toxicity

After integrating the output of the software tool, we obtained an overview of the toxicity profile of tetracycline (see Supplementary B for details), and the toxicity modeling suggests that the active target of tetracycline toxicity is related to immunotoxicity. These findings are consistent with previous reports of tetracycline-mediated toxicity in humans in the literature, and provide a basis for further systematic and in-depth studies of the toxic effects of tetracycline in humans.

### 3.2. Identification of Tetracycline-Induced Acute Pancreatitis Targets

**Figure 2:**
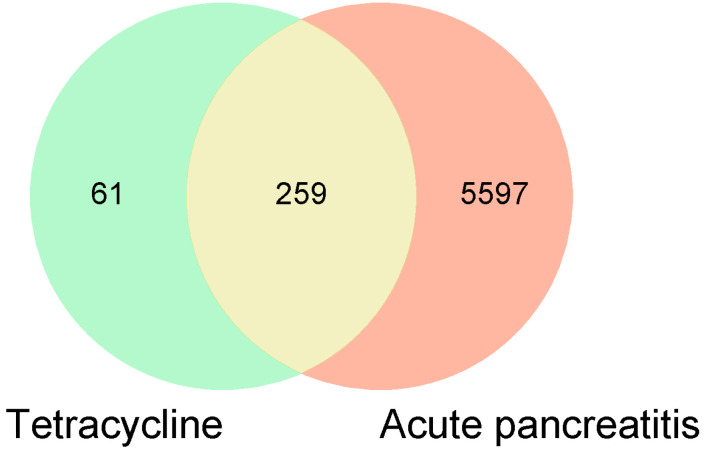
The intersection of 320 tetracycline clinical targets screened from the SwissTargetPrediction, SEA Search, and Super PRED databases with 5856 targets highly associated with acute pancreatitis identified through the GeneCards, Drugbank, and OMIM databases can be visualized by plotting the Venn diagrams. The overlap between the two datasets is significant. The overlap region between the two datasets clearly reveals 259 potential targets specifically associated with tetracycline-induced acute pancreatitis.

In this study, we first screened 320 tetracycline targets from the SwissTargetPrediction, Super-PRED, and GeneCards databases, and identified through the GeneCards, Drugbank, and OMIM databases a highly associated 5856 targets. The integration and deletion of these target sets yielded a total of 259 overlapping targets (see Supplementary C for detailed target names) as potential targets for tetracycline-induced acute pancreatitis (Figure 2). Figure 2 shows a Venn diagram illustrating the targets of tetracycline and acute pancreatitis. The 320 tetracycline targets screened from the SwissTargetPrediction, SEA Search, and Super-PRED databases were intersected with 5856 targets identified as highly relevant to acute pancreatitis through the GeneCards, Drugbank, and OMIM databases. Notably, the region of overlap between the two datasets revealed 259 potential targets specifically associated with tetracycline-induced acute pancreatitis.

### 3.3. Potential Targets and the Interaction Network of Essential Gene Acquisition

**Figure 3:**
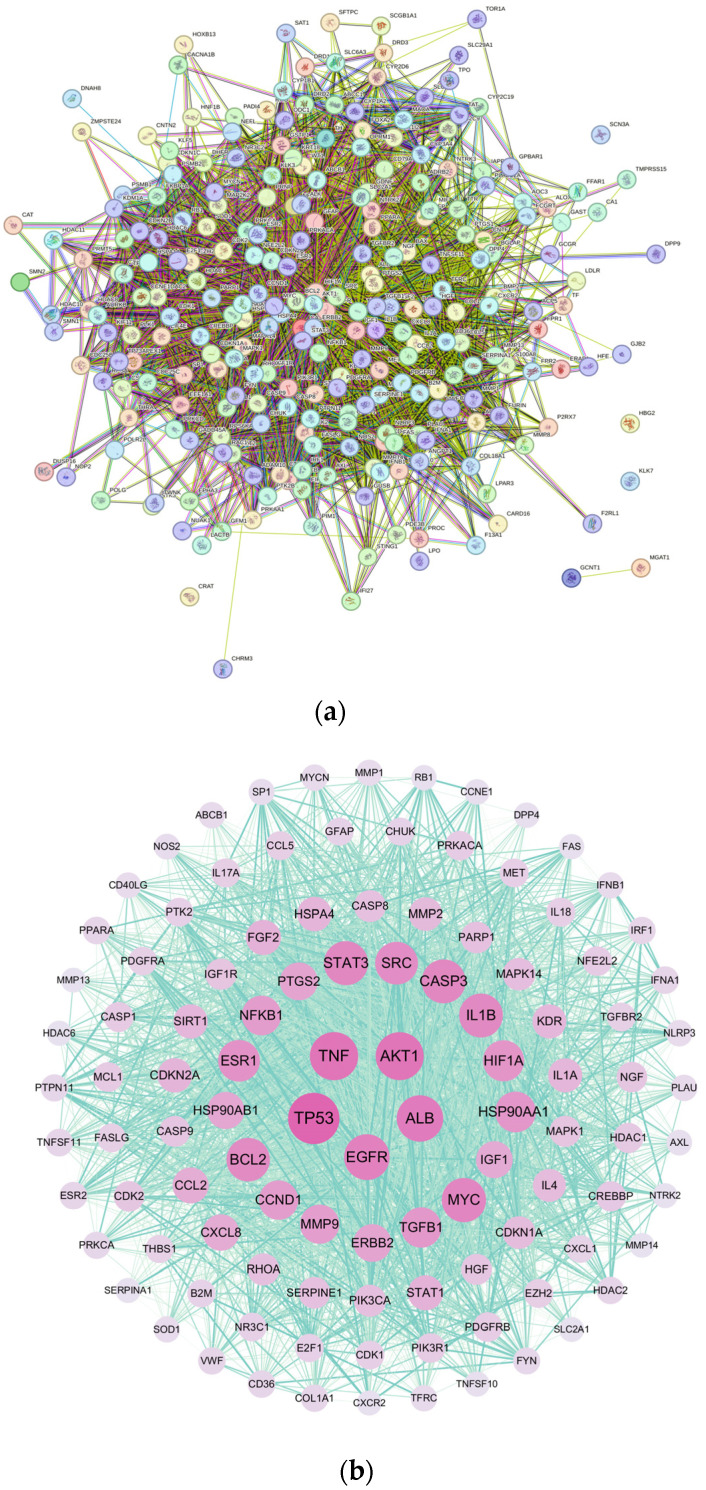
Protein–protein interaction (PPI) network construction and core target visualization for tetracycline-induced acute pancreatitis: (**a**) Original PPI network exported from String database, with different targets and their interactions distinguished by colors. (**b**) Visualization of selected targets in Cytoscape (version 3.10.1) software, where purple represents the intersection of tetracycline and acute pancreatitis targets, and green lines represent the interconnections between targets. In the network of (**b**), each node represents a gene, while the edges indicate their interactions. The size of the node is directly related to its degree, and the intensity of the color reflects the betweenness centrality of the node.

A PPI network was constructed using the STRING database, which contains a total of 259 nodes and 5237 edges. At the same time, we used Cytoscape (version 3.10.1) software to analyze the topological characteristics of network nodes, including degree centrality and betweenness, and to visualize them (Figure 3). Due to the large number of overlapping targets, we selected targets with a degree value of 40–175 to create a visually optimized protein–protein interaction map. The proteins in the middle of the network are more closely related to other proteins and may have potential targets. We mapped the target points into five layers according to the degree value. The larger the degree value, the closer the target point is to the center, that is, the closer its relationship with other targets. From the inside out, the first layer of target points has a rank value of 140–175, and the circle layer is red; the second layer of target points has a rank value of 100–139, and the circle layer is pink; the third layer of target points has a rank value of 70–99, with a purple-blue circle layer; the fourth layer of target points has a value of 55–69, with a sky-blue circle layer; and the fifth layer of target points is the scale, with a value of 40–54 and a cyan circle layer. The node size and color correspond to the respective degree value; the larger the node, the more vibrant it is and the higher the degree of representation. The thickness and color depth of the edges are directly proportional to their connection scores, with thicker and darker edges indicating higher connection scores.

Through network analysis, we identified 22 major targets of tetracycline-induced acute pancreatitis (Table 1). Notably, the top three targets based on the ranking value were TP53, TNF, and AKT1. These genes are widely recognized in current research to encode proteins that play important roles in a variety of cellular functions, including cell cycle regulation, DNA damage repair, apoptosis induction, and key roles in inflammatory responses and immune regulation.

**Figure 4:**
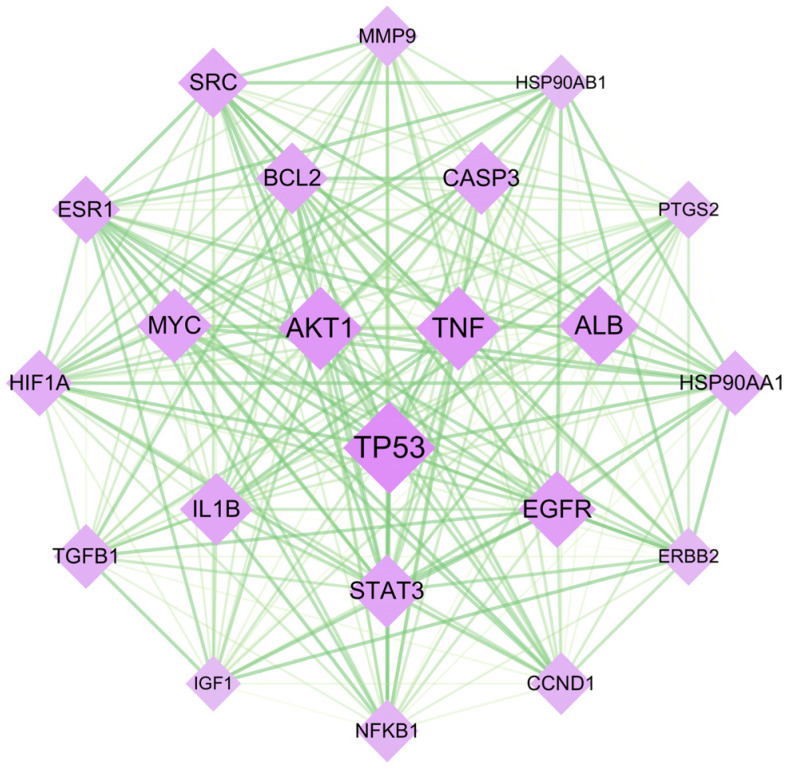
Protein–protein interaction (PPI) network diagram of 22 core targets. Each purple node represents a gene, while green lines indicate the interactions between them. The size of a node corresponds to its degree, with larger nodes indicating higher connectivity within the network. The intensity of the node’s color reflects its betweenness centrality, where darker colors denote greater importance as key mediators. The thickness and color intensity of the edges represent the strength of interactions between targets; thicker and darker lines signify stronger interactions.

The PPI network of the 22 core targets was constructed using Cytoscape (version 3.10.1) software to visually represent the interactions between the potential targets identified in this study (Figure 4). The node size and color correspond to the respective degree value, with larger and more vibrant nodes representing higher degrees. The thickness and color depth of the edges are directly proportional to their connection scores, with thicker and darker edges indicating higher connection scores.

The network was constructed based on the complex interactions of 22 core targets. This optimized network diagram provides a clear visual representation of the relationships and functional associations between these core targets. By analyzing the connection patterns and interactions within the network, it provides insights into the molecular interactions of tetracycline-induced pancreatic toxicity. Among these core targets, TP53, TNF, and AKT1 have the highest degree values, as can be seen from their larger font sizes and redder colors in the figure.

### 3.4. GO and KEGG Analysis of Potential Targets

**Figure 5:**
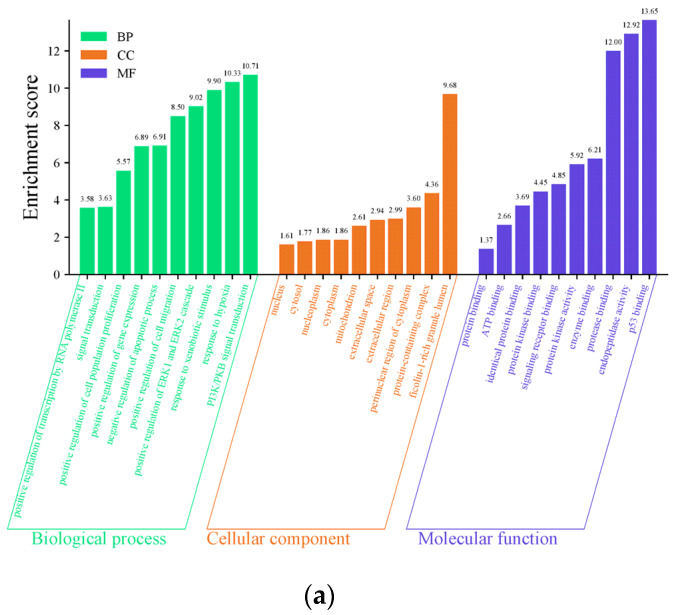

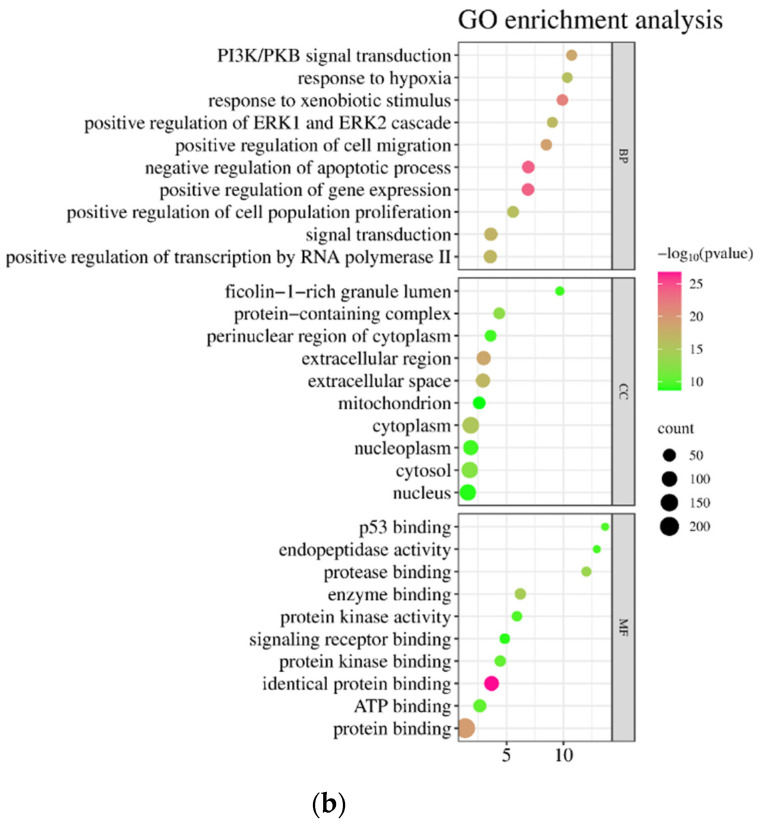
The top 10 enriched Gene Ontology (GO) terms for each category—biological process (BP), cellular component (CC), and molecular function (MF). (**a**) This histogram shows the top 10 enriched entries for each GO category (BP, CC, and MF). The FDR value reflects the statistical significance of the enrichment, with lower values indicating higher significance. The height of each bar corresponds to the gene count, reflecting the degree of enrichment in the corresponding category. These enriched terms highlight key biological processes, cellular components, and molecular functions that may be affected by tetracycline exposure. (**b**) The bubble plot presents the top 10 enriched pathways for the BP, CC, and MF categories. The bubble size reflects the number of genes associated with each pathway, representing the degree of enrichment, while the bubble color intensity corresponds to the statistical significance (measured by FDR value), with darker colors indicating higher significance.

We used the DAVID database to perform GO analysis on 259 potential targets, limiting the species to Homo sapiens. Our analysis yielded a total of 1200 GO entries, including 903 biological processes (BPs), 111 cellular components (CCs), and 186 molecular functions (MFs). Among these, 950 GO entries, including 714 biological processes, 96 cellular components, and 140 molecular functions, were statistically significant (*p* < 0.05). The GO terms were ranked according to the false discovery rate (FDR) value, and the top 10 terms with the lowest FDR values in BPs, CCs, and MFs were selected and visualized in an enrichment analysis diagram (Figure 5).

**Figure 6:**
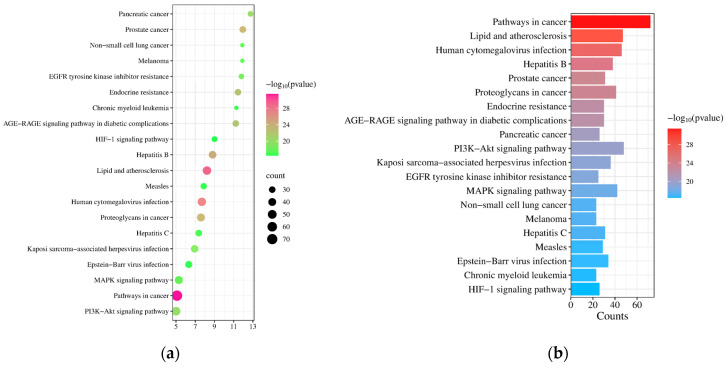
The top 20 enriched Kyoto Encyclopedia of Genes and Genomes (KEGG) pathways. (**a**) Each bubble represents a specific pathway, where the bubble size indicates the number of genes enriched in the pathway. The intensity of the bubble color indicates the significance of the enrichment, with darker red indicating higher significance. (**b**) The histogram shows the frequency and significance of the top 20 pathway enrichments. The length of each bar corresponds to the gene count and indicates the enrichment score. The color of each bar corresponds to the significance level, with redder colors indicating higher significance.

In addition, we used the DAVID database to perform a KEGG analysis on these 259 potential targets to determine their involvement in specific signal pathways. Among the 181 signal pathways enriched in total, we sorted them in ascending order of FDR value and generated a bubble chart with significance statistics and a classification histogram (Figure 6) to visually represent the top 20 KEGG signal pathways. Our research results show that tetracycline may induce acute pancreatitis mainly through lipid-related signaling pathways such as atherosclerosis, AGE-RAGE, PI3K-Akt, MAPK, and HIF-1.

### 3.5. Molecular Docking of Tetracycline and Core Target Proteins in Acute Pancreatitis

**Figure 7:**
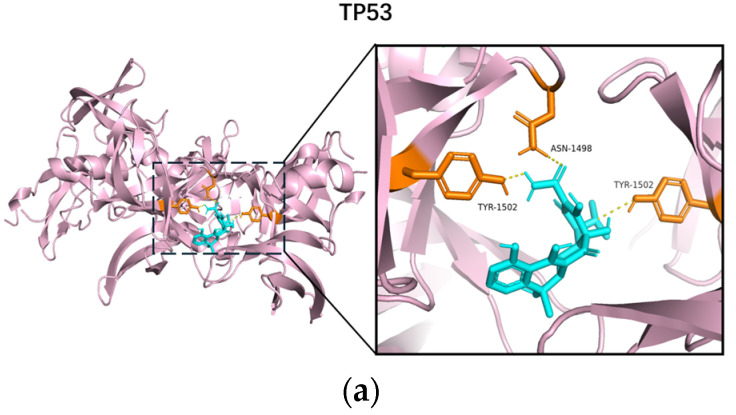

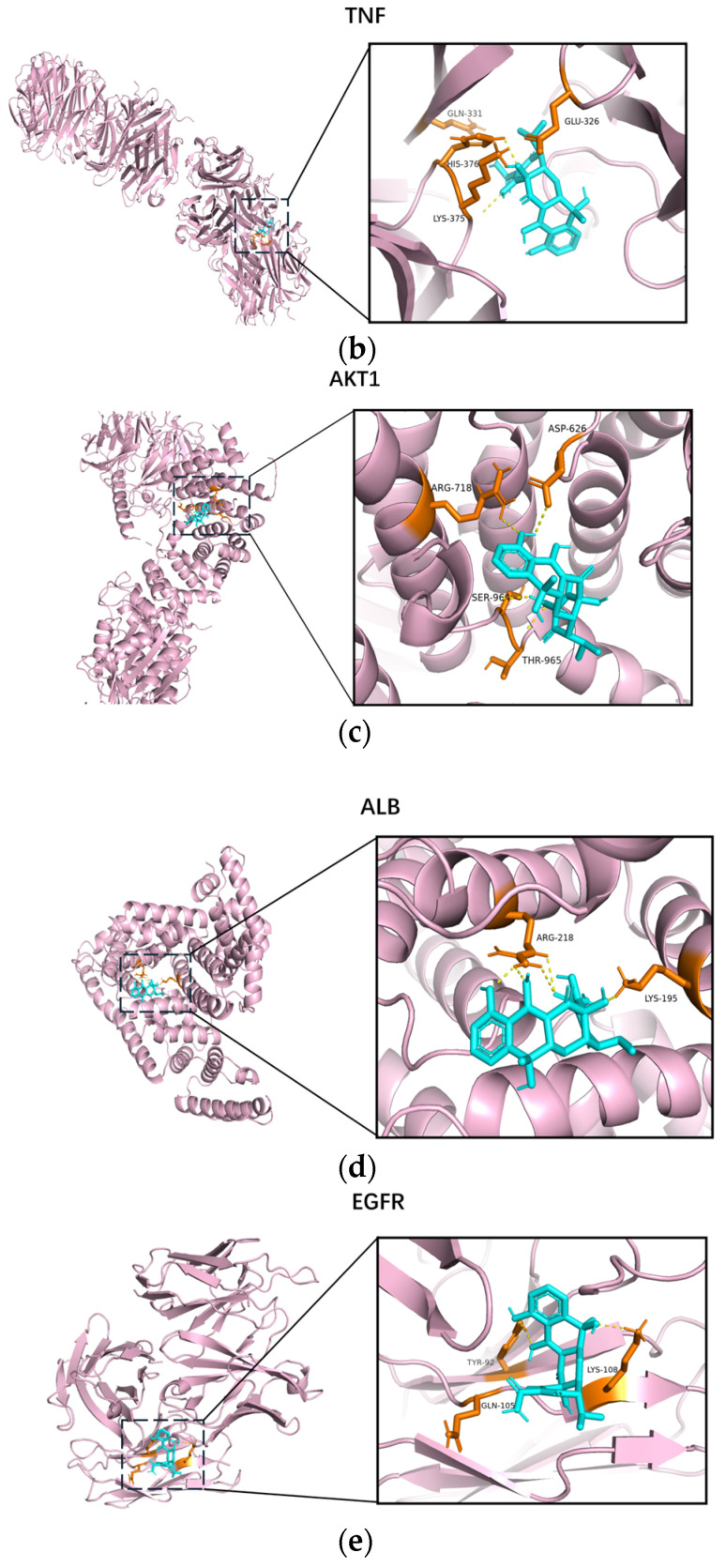
Molecular docking results of the lowest binding energy in each target protein with tetracycline. (**a**) tetracycline and TP53; (**b**) tetracycline and TNF; (**c**) tetracycline and AKT1; (**d**) tetracycline and ALB; (**e**) tetracycline and EGFR.

The interactions between tetracycline and the 22 core target genes were studied through molecular docking analysis (Figure 7). This indicates a strong affinity between the compound and the target. It is worth noting that all 22 core target proteins and tetracycline exhibit strong binding affinity, with binding energies <0, indicating that tetracycline can spontaneously bind to these core target proteins and play an important role in the molecular mechanism of tetracycline-induced pancreatic toxicology. Due to the length limitations of this article, we only include the molecular docking result graphs of the five most core target proteins. The remaining result graphs and data on the lowest binding energy of molecular docking can be viewed in the Supplementary Materials.

## 4. Discussion

In our study, after applying the network assessment tool, we systematically screened 259 potential targets related to tetracycline-induced acute pancreatitis using the SwissTargetPrediction, SEA Search, STITCH, Super–PRED, GeneCards, OMIM, and TTD databases. Based on the STRING database and Cytoscape, we constructed an interaction network of potential target genes and extracted 22 key nodes, including TP53, TNF, AKT1, ALB, and EGFR, which are core targets in the context of tetracycline-induced acute pancreatitis.

Regarding TP53, also known as the “guardian of the genome”, it encodes the p53 protein, an important tumor suppressor protein that also plays an important role in human homeostasis in cell cycle regulation, control of apoptosis, and maintenance of genomic stability [21,22]. p53 is associated with anti-inflammatory activity by signal transducer and activator of transcription 3 (STAT3) to regulate inflammation [23]. STAT3 acts downstream of interleukin (IL-6), which has both pro-inflammatory and anti-inflammatory potential in acute inflammatory responses [24,25].

TNF can be divided into TNF-α and TNF-β. As a powerful pro-inflammatory cytokine, TNF can induce cell death and thus indirectly promote the inflammatory response. In addition, it can also induce the expression of inflammatory genes, thereby directly driving inflammation [26]. Under normal circumstances, tetracycline can inhibit the synthesis of the pro-inflammatory factor TNF-α or its mRNA synthesis, thereby exerting an anti-inflammatory effect [27]. However, in the special microenvironment of the pancreas, the body’s inflammatory balance is very delicate. When tetracycline is used, it may disrupt the original balance, leading to a disorder of other compensatory mechanisms, which in turn indirectly causes adverse effects on the pancreas and promotes the pathogenesis of acute pancreatitis.

The protein encoded by the AKT1 gene is a serine/threonine kinase that belongs to the protein kinase B (PKB) family. It is a key node in the intracellular signal transduction pathway, capable of receiving signals from upstream signal molecules and transmitting them to downstream target molecules [28]. When cells are stimulated by harmful external factors, the upstream signal pathway may be abnormally activated or inhibited, and interference with the function of the AKT1 gene and its protein product will affect the signal transduction network. Abnormal AKT1 signaling can cause metabolic disorders in pancreatic acinar cells, making pancreatic cells more sensitive to damaging factors such as abnormal activation of trypsinogen and oxidative stress [29]. The AKT1 protein may also regulate the activity of key molecules in inflammatory-related signaling pathways. Dysfunction can promote the excessive release of inflammatory factors, exacerbate inflammatory responses, and promote the development of pancreatitis [30].

In an inflammatory state, serum ALB levels are usually reduced, mainly because the levels of inflammatory factors (such as IL-6) and tumor necrosis factors (such as TNF-α) are elevated and directly affect the synthesis of ALB by acting on liver cells [31]. At the same time, during the inflammatory process, the body is in a state of “stress”, with an increased metabolic rate and increased energy requirements [32]. In order to meet the body’s needs, the body breaks down albumin to provide amino acids for processes such as gluconeogenesis, providing energy for immune cells and other cells in the inflammatory response, which in turn aggravates inflammation and leads to the development of acute pancreatitis.

EGFR, a member of the tyrosine kinase receptor family, is involved in a variety of physiological processes, including cell proliferation, differentiation, adhesion, migration, and apoptosis [30,33]. In recent years, studies have shown that EGFR signaling plays a key role in the activation and function of macrophages and is essential for the production of inflammatory chemokines in vivo [34]. In addition, proper EGFR signaling can help maintain cellular homeostasis and prevent excessive inflammatory responses. In the pathogenesis of acute pancreatitis, when certain factors (such as pancreatic juice reflux, abnormal activation of pancreatic enzymes, etc.) occur, it may cause changes in the local microenvironment, which can interfere with normal EGFR signaling and cause it to lose precise regulation of the inflammatory response. On the one hand, abnormal EGFR signaling may overactivate macrophages, promote the release of a large number of inflammatory chemokines, and trigger an inflammatory cascade reaction [35]. On the other hand, cell homeostasis is disrupted, and inflammation cannot be effectively suppressed, leading to the exacerbation of inflammation in pancreatic tissue. Pathological changes such as edema, bleeding, and necrosis appear one after the other, ultimately leading to the development of acute pancreatitis [36].

According to the results of KEGG pathway enrichment analysis, tetracycline may induce acute pancreatitis by affecting lipid and atherosclerosis, AGE-RAGE, PI3K-Akt, MAPK, HIF-1, and other signaling pathways. In the lipid and atherosclerosis signaling pathway, tetracycline may affect the activity of key enzymes or transport proteins in lipid metabolism, thereby changing the lipid metabolic process in pancreatic cells. It may inhibit enzymes that contribute to normal lipid metabolism, causing lipids to accumulate in pancreatic cells and the number of lipid droplets to increase [37,38]. At the same time, abnormal lipid metabolism can trigger an inflammatory response, activate related signal pathways, and increase the release of inflammatory factors [39], ultimately leading to the development of acute pancreatitis. In terms of the AGE-RAGE pathway, the mechanism by which tetracycline induces acute pancreatitis may be to increase the production of advanced glycation end-products (AGEs) or enhance the binding of AGEs to the receptor (RAGE). When this pathway is activated, it triggers a series of cascading reactions, activates nuclear factor-κB (NF-κB), and in turn increases inflammatory factors such as interleukin-1β (IL-1β) and TNF-α, which cause damage to pancreatic tissue [40]. The PI3K-Akt signaling pathway plays a key role in processes such as cell survival, proliferation, and metabolism [41]. Tetracycline may interfere with the function of key molecules in this pathway, affecting the production of phosphatidylinositol-3,4,5-triphosphate (PIP3) or the phosphorylation and activation of Akt protein, thereby disrupting the metabolism of pancreatic cells, altering the sensitivity of pancreatic cells to apoptotic signals, promoting cell damage and inflammatory responses [42], and ultimately inducing acute pancreatitis. For the MAPK signaling pathway, it is very likely that tetracycline affects upstream signaling, leading to the phosphorylation and activation of MAPK members, the activation of transcription factors such as activator protein-1 (AP-1), the regulation of gene expression, the production of inflammatory factors, and effects on pancreatic cell function, triggering inflammation and damage [43], which in turn leads to acute pancreatitis. In addition, an abnormal HIF-1 pathway can trigger an inflammatory response [44]. Tetracycline may abnormally activate this pathway under aerobic conditions through specific regulatory mechanisms, causing a stable accumulation of the hypoxia-inducible factor-1α (HIF-1α) protein, which initiates the transcription of inflammation-related genes, promotes the infiltration of inflammatory cells and the release of inflammatory factors, and affects the energy metabolism of pancreatic cells, triggering inflammation and damage [45] and thereby inducing acute pancreatitis.

In addition to providing the molecular mechanism of tetracycline-induced acute pancreatitis, this study also proposes a network toxicology strategy for the rapid study of the toxicity of potential drugs. Traditional toxicology studies often use animal models as a basis, while experimental techniques in pathology and immunology are used to identify the toxic targets of drug factors. Although these reductionist approaches are invaluable, they face certain limitations, especially in keeping up with the rapid emergence of potential chemical toxicants in drugs. First, the length and cost of animal experiments limits the breadth of toxicology studies. This hinders the assessment of the large number of unstudied drugs that have proliferated as a result of industrial development. Second, due to species differences in physiology, genetics, and molecular pathways, animal models often do not perfectly recapitulate human responses, providing only a vague interpretation of the relevant drug’s toxicity mechanism.

On the other hand, network toxicology analysis, which utilizes the latest advances in bioinformatics, genomics, and big data analysis of databases, can quickly and comprehensively map the complex molecular relationships linking new toxicants and pathological endpoints. By modeling biomolecular networks across multiple biochemical scales, network toxicology can reveal common mechanisms of new chemical substances and prioritize potential targets and core targets that mediate toxic phenotypes. In addition, molecular docking techniques have become the most critical and widely used method for elucidating biological and molecular mechanisms, especially in the process of predicting and simulating complex structures at the molecular or even atomic level. The use of network-based toxicology and molecular docking paradigms will likely expand the efficiency, depth, and predictive accuracy of toxicological screening, facilitating the assessment of a large number of understudied drugs with unexpected toxicities.

While this study provides valuable insights into the molecular mechanisms underlying tetracycline-induced acute pancreatitis, certain limitations must be addressed. The reliance on publicly available databases, such as STRING and DAVID, may introduce variability due to incomplete or outdated data, potentially impacting the comprehensiveness of target and pathway identification. Although network toxicology and molecular docking serve as powerful predictive tools, their findings remain theoretical and necessitate experimental validation through in vitro assays, in vivo models, and clinical trials to confirm their biological relevance. Moreover, the study’s focus on tetracycline-induced acute pancreatitis may limit the generalizability of its findings to other drug-induced or multifactorial cases of pancreatitis. Additionally, the computational models used in molecular docking, including grid parameters and scoring functions, may influence the accuracy of predicted binding interactions, necessitating cross-validation with alternative tools and experimental results to enhance the robustness of the conclusions. Addressing these limitations in future research will enable a deeper understanding of tetracycline’s effects on acute pancreatitis and facilitate the development of targeted prevention and treatment strategies.

## 5. Conclusions

This study sheds light on the molecular mechanisms underlying tetracycline-induced acute pancreatitis by identifying critical proteins and pathways implicated in its pathogenesis. Specifically, tetracycline interacts with key inflammation-related proteins such as TP53, TNF, and AKT1, which regulate processes including pancreatic cell apoptosis and proliferation. Functional enrichment analysis pinpointed significant pathways, including PI3K-Akt, MAPK, and AGE-RAGE, suggesting tetracycline’s potential to disrupt cellular homeostasis and exacerbate inflammatory responses.

Molecular docking revealed strong binding affinities between tetracycline and these core targets, strengthening the hypothesis that these interactions promote the initiation and progression of inflammatory cascades. Notably, TP53 exhibited the strongest binding affinity, further emphasizing its role as a central mediator in tetracycline-induced acute pancreatitis.

The integration of network toxicology and molecular docking in this study establishes a robust methodological framework for investigating drug-induced toxicities. These findings provide theoretical insights into tetracycline-associated inflammation and lay a foundation for developing targeted interventions to mitigate its adverse effects. Future research should focus on validating these mechanisms through in vitro and in vivo studies, with an emphasis on identifying therapeutic targets to prevent or treat tetracycline-induced acute pancreatitis effectively.

## Figures and Tables

**Table 1:** Ranked intersection targets based on degree, betweenness, and closeness values for the top 22 key targets associated with tetracycline-induced acute pancreatitis.

	Name	Degree	Betweenness	Closeness
1	TP53	175	5772.26	0.7652439
2	TNF	159	2887.652	0.73177844
3	AKT1	158	2908.7302	0.72965115
4	ALB	150	4823.652	0.7130682
5	EGFR	145	1915.3193	0.69529086
6	MYC	139	1498.9303	0.6839237
7	STAT3	138	1169.8619	0.68767124
8	CASP3	137	1208.9492	0.6857923
9	IL1B	136	1645.6488	0.6820652
10	BCL2	134	1067.7808	0.6783784
11	SRC	131	1757.402	0.6657825
12	ESR1	126	1686.3923	0.66402113
13	HIF1A	123	1050.1356	0.6570681
14	HSP90AA1	121	1374.9637	0.6536458
15	TGFB1	119	986.43243	0.6485788
16	CCND1	114	670.39246	0.6403061
17	NFKB1	114	527.567	0.64194375
18	MMP9	113	500.38266	0.64194375
19	PTGS2	106	867.17944	0.62907267
20	ERBB2	106	618.3276	0.62593514
21	HSP90AB1	103	696.5803	0.6243781
22	IGF1	100	470.61935	0.61975306

## Data Availability

The data that support the findings of this study are available from the corresponding author upon reasonable request.

## Supplementary Materials

The following are available online at https://www.mdpi.com/article/10.3390/toxics12120929/s1, Supplementary A: The name of the detailed database used in this study and the corresponding URL used. Supplementary B: Overview of the toxicity profile of tetracycline. Supplementary C: The intersection of tetracycline and acute pancreatitis target collection; Figure S1: Molecular docking results of the lowest binding energy for each target protein with tetracycline; Table S1: Targets of tetracycline; Table S2: Targets of acute pancreatitis; Table S3: GO enrichment analysis results; Table S4: KEGG enrichment analysis results; Table S5: Minimum binding energy data for docking of core target molecules.
