# Supplementary material for: Network Toxicology and Molecular Docking Analysis of Tetracycline-Induced Acute Pancreatitis: Unveiling Core Mechanisms and Targets
Source: Toxics. 2024 Dec 21;12(12):929. doi: 10.3390/toxics12120929 (PMC11679059; doi:10.3390/toxics12120929)
Supplement: Supplementary file 1 [file toxics-12-00929-s001.zip › Supplementary materials/Figure S1.pdf]

MYC

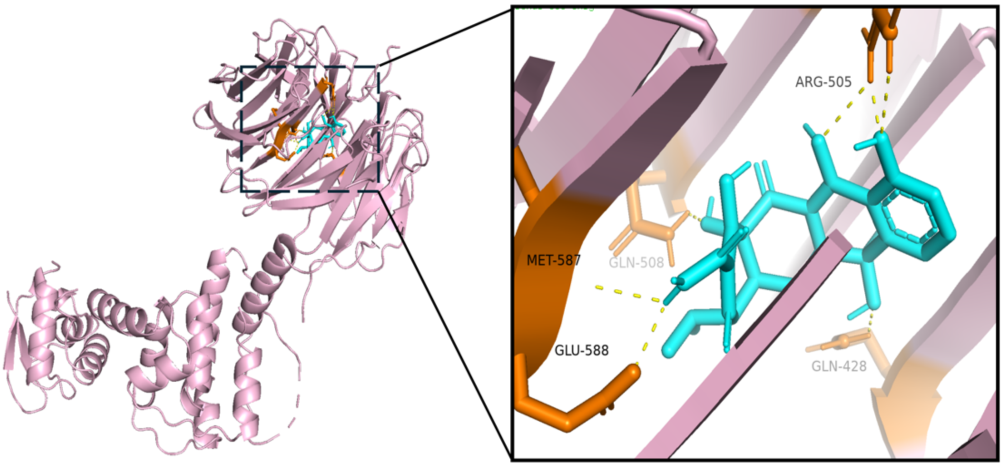

STAT3

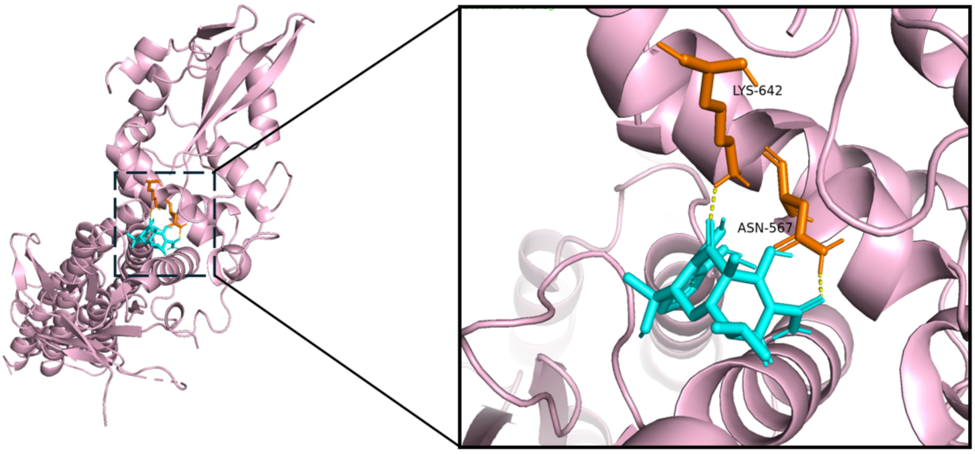

CASP3

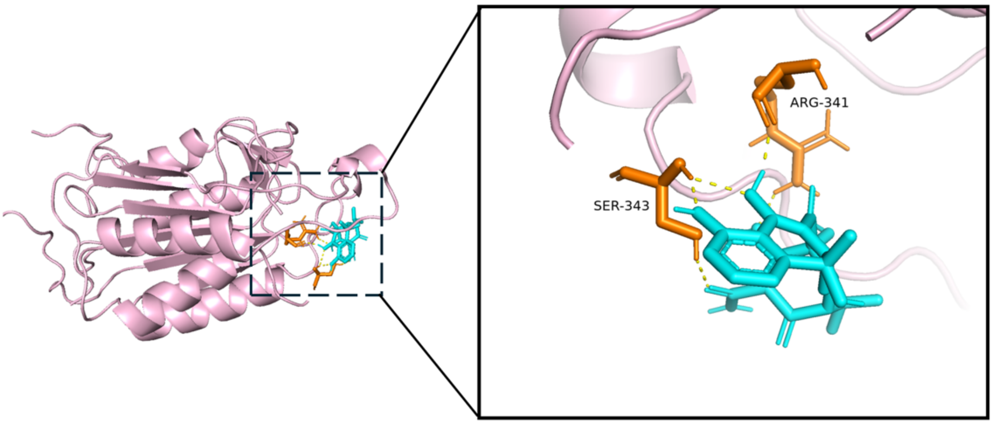

IL-1β

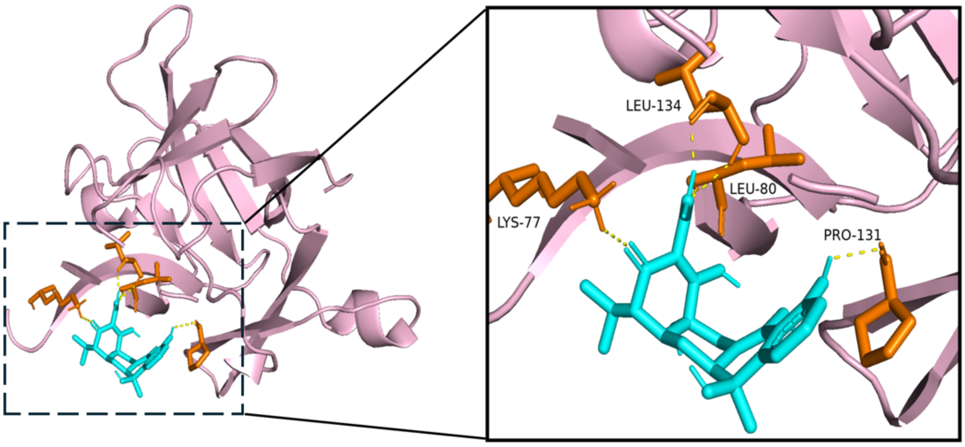

BCL2

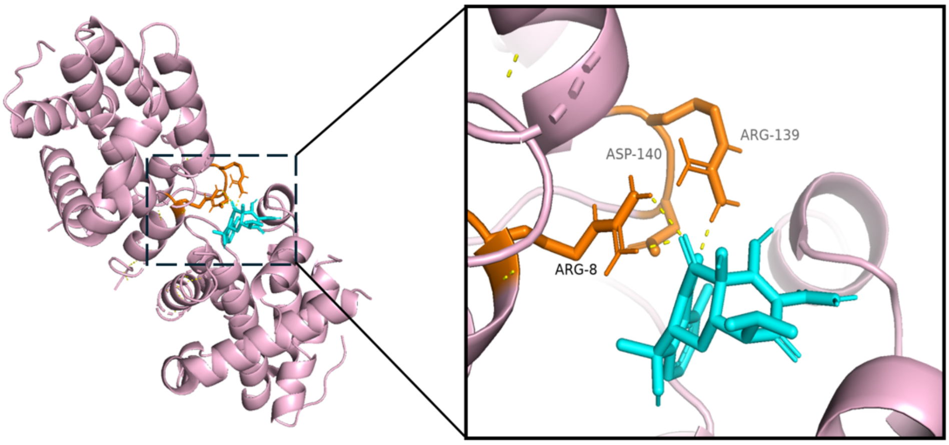

SRC

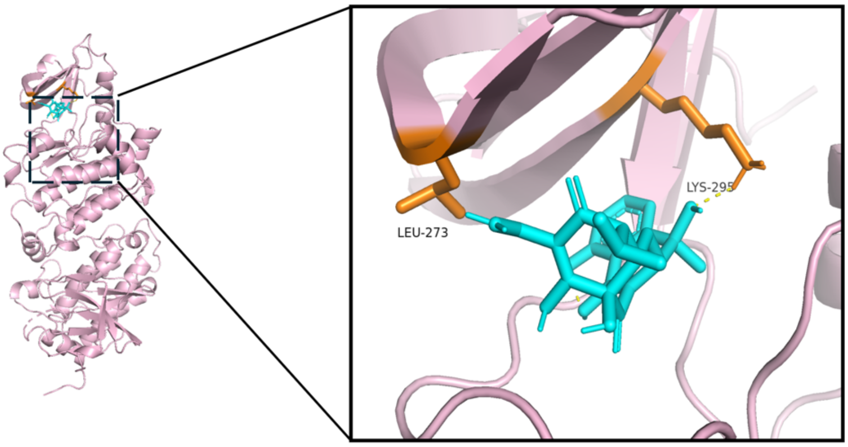

ESR1

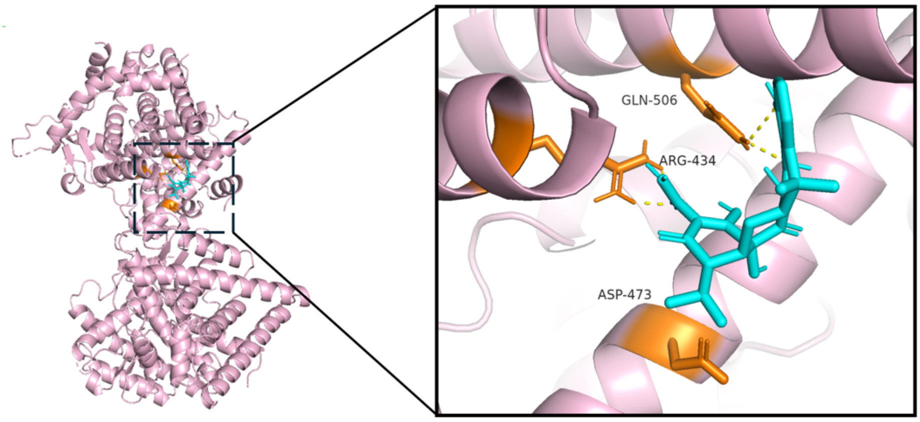

HIF1A

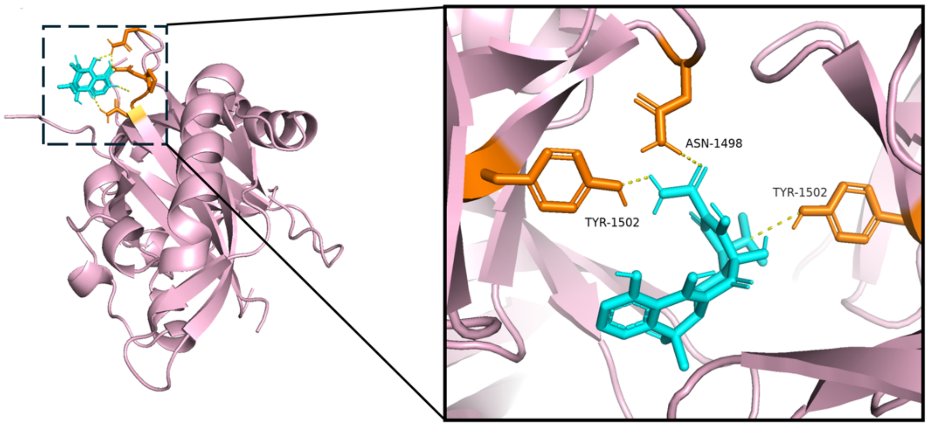

HSP90AA1

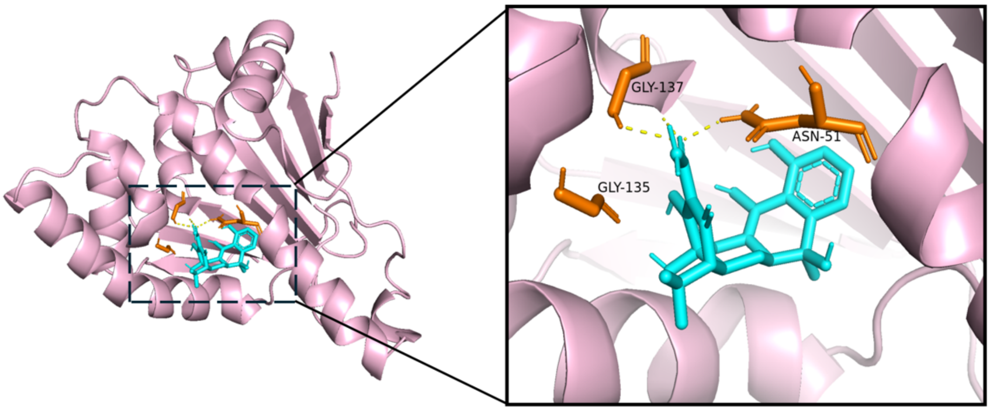

TGFB1

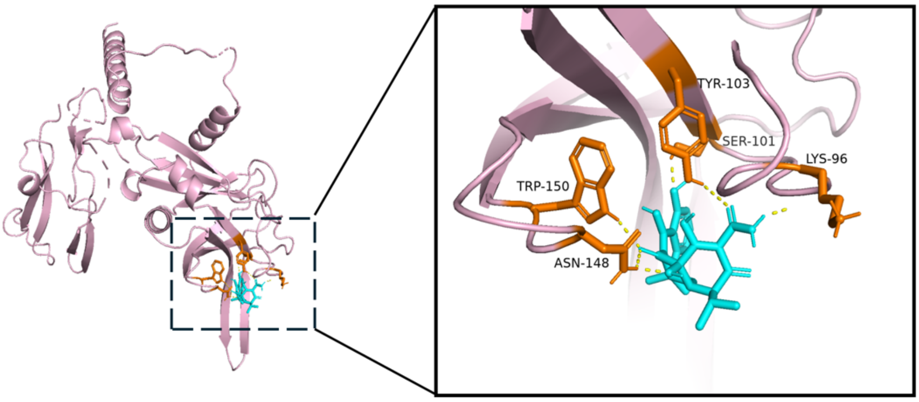

CCND1

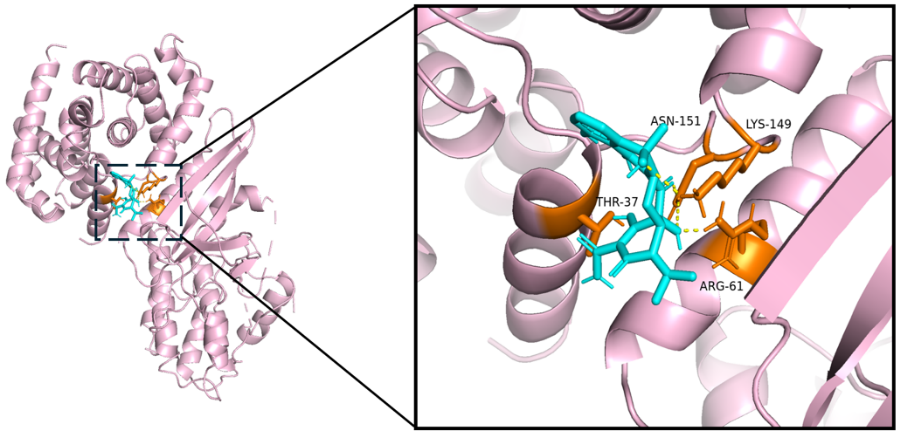

NFκB1

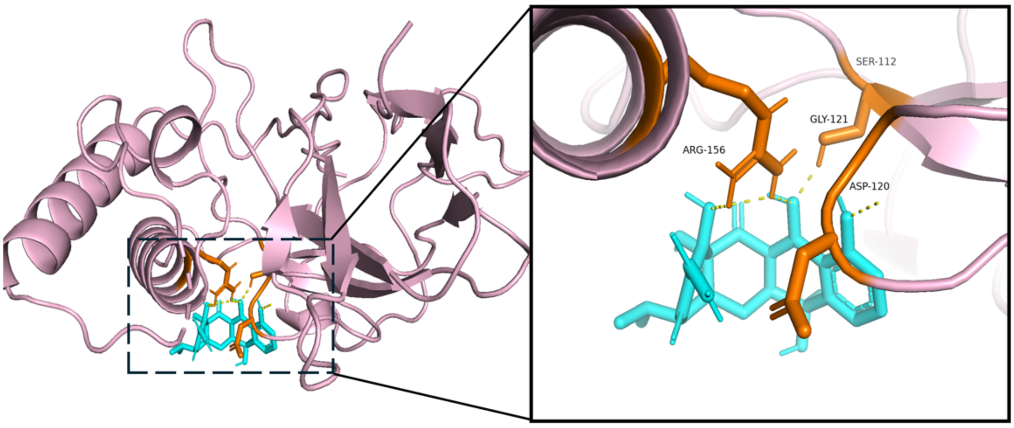

MMP9

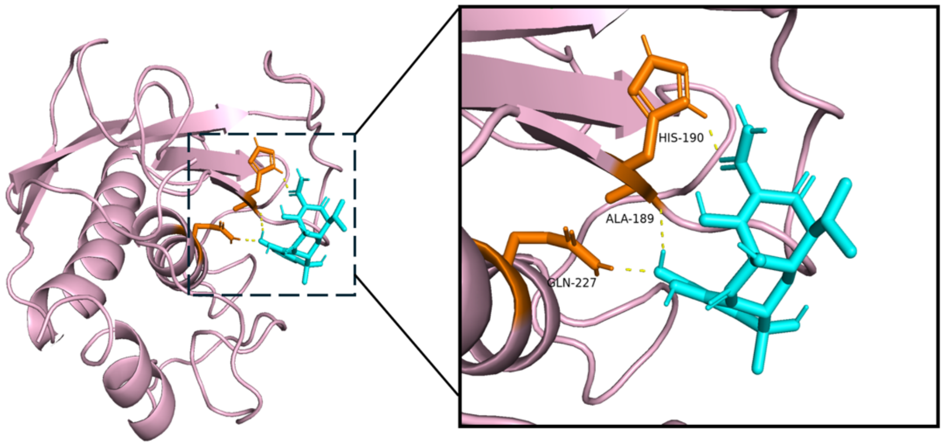

PTGS2

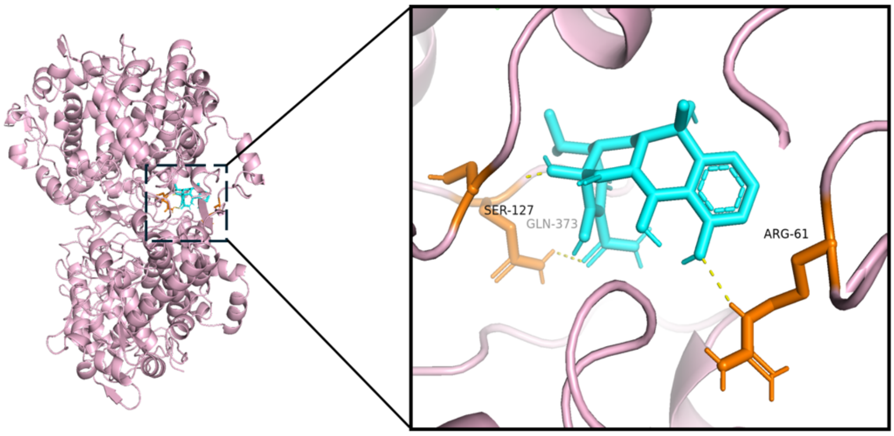

ERBB2

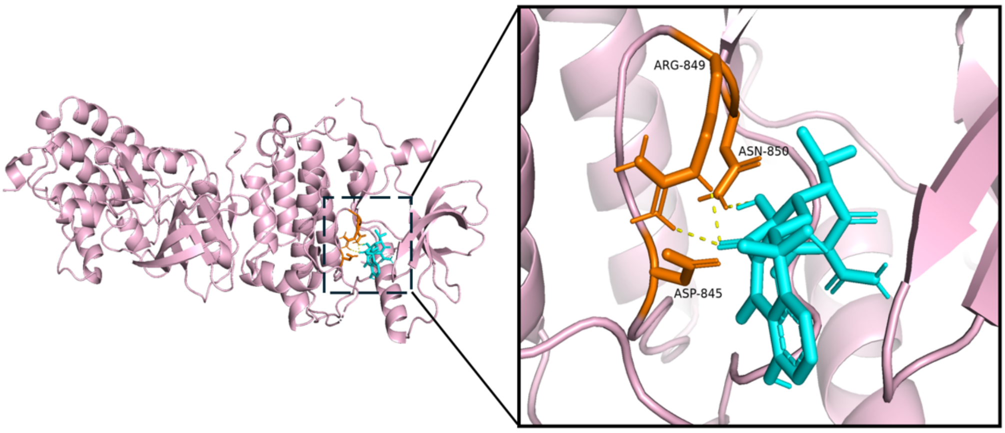

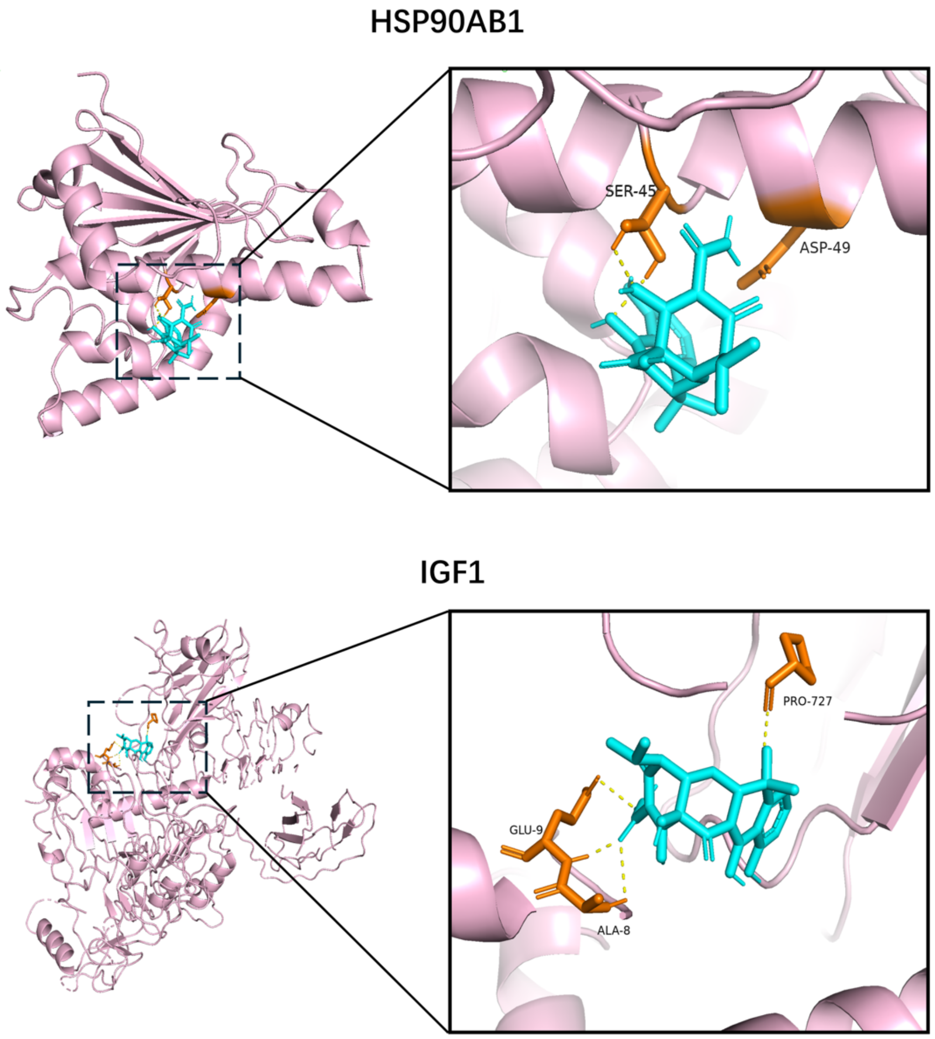

Figure S1. Molecular docking results of the lowest binding energy for each target protein with tetracycline. The figure includes docking interactions of tetracycline with the following proteins: MYC, STAT3, CASP3, IL1B, BCL2, SRC, ESR1, HIF1A, HSP90AA1, TGFB1, CCND1, NFKB1, MMP9, PTGS2, ERBB2, HSP90AB1, and IGF1.
